# Assessment of Alcohol-Based Hand Sanitizers for Long-Term Use, Formulated with Addition of Natural Ingredients in Comparison to WHO Formulation 1

**DOI:** 10.3390/pharmaceutics13040571

**Published:** 2021-04-17

**Authors:** Francesca Fallica, Chiara Leonardi, Valeria Toscano, Debora Santonocito, Paola Leonardi, Carmelo Puglia

**Affiliations:** 1 Department of Drug and Health Science, University of Catania, Viale Andrea Doria 6, 95125 Catania, Italy; francescafallica19@gmail.com (F.F.); debora.santonocito@unict.it (D.S.); 2 Department of Biological, Geological and Environmental Sciences, University of Catania, Via Androne 81, 95124 Catania, Italy; chiara.leonardi@unict.it; 3 Research & Develepment Department, PLB Cosmetici, Via Collodi 1, 95024 Acireale, Italy; paola.leonardi@biocosme.it; 4 National Research Council, Institute for Agricultural and Forest Systems in the Mediterranean, Via Empedocle 58, 95128 Catania, Italy; valeria.toscano@cnr.it

**Keywords:** COVID-19, gel formulation, viscosity enhancer, skin hydration, biocide efficiency, hedonic values

## Abstract

During the spread of COVID-19, many laboratories used the “*Formulation 1*” proposed by the World Health Organization to prepare hand sanitizers. Taking into consideration its ingredients and the prolonged use of hand sanitizers, “*Formulation 1*” (P1) was compared with two gel formulations (P2 and P3) prepared with the addition of natural emollients and two different viscosity enhancers to define their chemical–physical stability, biocidal efficacy, and in vivo acceptability and tolerability. P1 resulted in the most efficient biocide but was poorly tolerated by the skin and not acceptable in volunteer hedonic evaluation, especially in terms of irritation and drying effect, with an expectable reduction in the compliance. Moreover, its liquid formulation is unpractical and can cause ethanol evaporation. P2 and P3 proved to be both good products regarding pH and alcohol strength values. However, in terms of viscosity, texture, ease of use, and application, P3 seemed to be a better gel product than P2. Moreover, they were well tolerated by the skin, increasing the hydration of the stratum corneum, due to the addition of *Calendula officinalis* and Aloe vera. Despite a lower ethanol concentration than P1, P2 and P3 also showed a good biocide efficiency, with better results in P2. In conclusion, these gel formulations proved to be more convenient for long-term use with a good balance between efficacy, safety, and compatibility with the skin.

## 1. Introduction

Nowadays, hand hygiene is considered a measure of extraordinary importance in the control of disease and cannot be underemphasized. If the scientific interest around hand hygiene in healthcare-associated infections (HCAIs), and the pivotal role of healthcare workers’ (HCWs) hands is old news, the concern about the act of handwashing in daily life increased during the spreading of SARS-CoV in 2003 and recently during the emergence of COVID-19. For both these diseases, it was observed that transmission is possible in the form of aerosol and fomites, and the virus can remain viable and infectious in aerosols for hours and on surfaces up to days, depending on the inoculum shed [1]. Moreover, in a period in which the vaccine is not yet available for everyone and still studies about the nature and contagiousness of the virus are being conducted, the act of handwashing has become of relevant importance to reduce transmission.

The alcohol-based hand sanitizers (ABHSs) are defined by the World Health Organization (WHO) as “an alcohol-containing preparation (liquid, gel or foam) for application to the hands to inactivate microorganisms and/or temporarily suppress their growth” and are considered useful in both hospital and community settings [2]. Indeed, the alcohol-based hand rubs (ABHRs) are the most effective and convenient infection preventive measure [3], above all because handwashing facilities are not readily available at work or public places [4].

When the COVID-19 pandemic spread worldwide, the use of alcohol-based hand rubs (ABHRs) increased exponentially, causing a lack of sanitizers available in the market. To respond to the severe shortage in hand sanitizers, pharmaceutical companies and cosmetic industries, breweries, and perfumeries have started, in an unprecedented move, to produce hand sanitizers [5]. Consumer habits and patterns are rapidly reshaping under the COVID-19 crisis and the unprecedented demand for hand sanitizers is likely to remain as the “new normal” for an extended period of time [6].

ABHSs mostly contain different types of alcohol such as isopropanol, ethanol, n-propanol, or a mixture of them in a recommended concentration of 60–85% by volume [7]. Notably, higher than these concentrations are also paradoxically less potent because proteins are not denatured easily without the presence of water [8]. In fact, solutions containing concentrations of alcohol >90% obtained a minor vitality reduction of bacteria and fungi in different studies [9]. Many studies showed that ABHSs in the dosage forms of gels and foams are more widely accepted by consumers, compared to liquid, especially in terms of handleability and low risk of spillage, although the latter left a high clean feeling and took a shorter time to dry [10]. Moreover, gel-based formulations reduce the evaporation rate of alcohol and help alcohol to spread and penetrate through contaminating organisms [11]. Thus, they can be considered the best products both in terms of users’ compliance and stability.

Gels can be obtained by incorporating different types of viscosity enhancer excipients that were listed [12], during the COVID-19 emergency, for the preparations of hydroalcoholic gels, by the Italian Society of Compounding Pharmacists (SIFAP); this list includes carbomer, hydroxypropyl cellulose (HPC), hydroxypropyl methylcellulose (HPMC), sodium carboxymethyl cellulose (CMC), hydroxyethyl cellulose (HEC). Xanthan gum (XG), not included in this list, is a viscosity enhancer that has received great attention from both researchers and manufacturers due to its nontoxicity, biocompatibility, biodegradability, and acceptable cost, which permit its use in various applications [13]. Indeed, it is a thickening agent, becoming more commonly used in cosmetic and pharmaceutical industries.

However, frequent application of hand hygiene products can be the cause of skin reaction and irritant contact dermatitis (ICD) [14] that are actually really common [15] and result in skin irritation, skin dryness, enlargement of pores, thus making sensitive skin more susceptible to infection [16].

The adverse effects caused by sanitizers can be easily prevented by selecting products that have a good balance between effectiveness, safety, and compatibility with all skin types [17]. For example, the prevention of ICD is possible by selection of a low-irritating hand rub, which contains emollients [18]. Glycerin is the most common humectant used in hand sanitizers and other cosmetic products; the studies in [4,19] showed that incorporation of glycerin in hand rubs promotes hand hydration to an extent that is directly proportional to its concentration in the formulation. The major issue concerning the use of glycerol is the fact that it can lower the bactericidal activity of ABHRs when used at a concentration of 1.45% (*v*/*v*), as shown in a study by Suchomel et al. [20]. Thus, although the WHO ethanol-based hand rub (EBHR) formulation contains 1.45% glycerol, reducing glycerol content to concentrations of 0.50−0.73% has been proposed as the best compromise in maintaining antimicrobial activity while still offering the needed skin protection [21].

Recently, the increasing consumer interest in natural products has suggested adding Aloe vera (*Aloe barbadensis* Mill.) gel as an emollient in hand sanitizers [22]. Aloe vera is an herbaceous perennial plant that belongs to the *Aloaceae* family and has been used for many centuries for its curative and therapeutic properties [23]. A study by Dal’Belo et al. [24], in which the moisturizing effects of cosmetic formulations containing different concentrations of lyophilized Aloe vera gel were studied, showed that formulations with higher concentrations (0.25% *w*/*w* and 0.5% *w*/*w*) increased the water content of the stratum corneum after a single application. The same study stated, furthermore, that Aloe vera extract improves skin moisture by a humectant mechanism. Moreover, Aloe vera has antimicrobial properties so it may increase the activity of sanitizers [22], but, until now, no study has demonstrated this synergistic action. Similar to Aloe vera, *Calendula officinalis* (L.) extract also has antifungal, anti-inflammatory, and antibacterial properties that might make it useful in healing wounds, soothing eczema, and relieving diaper rash, and it is also used as an antiseptic [25]. In fact, through web research findings, *Calendula officinalis* extract and oil have been shown to be used more commonly as excipients in hand sanitizer formulations.

Fragrances are another component that is usually added to alcohol-based hand sanitizers in order to adjust the smell produced by alcohol or other components. According to the WHO, alcohol-based hand-rub preparations with strong fragrances may be poorly tolerated by those HCWs with respiratory allergies. In fact, the most common causes of contact allergies are fragrances and preservatives, with emulsifiers being less common [26]. Consequently, consideration should be given to selecting a product with mild or no added fragrances [14]. A valid alternative to fragrances can be adding essential oils to ABHRs because in addition to being generally considered as safe at low concentrations, indeed they are used as food additives, they also carry out antimicrobial and antioxidant action [27].

The aim of this work was to produce and evaluate two different gel formulations of ABHS which, due to the addition of natural emollients and essential oils, could have antimicrobial action and, at the same time, result in acceptable long-term usage in terms of impact on the skin and hedonistic needs of consumers and compare them to WHO Formulation 1 published in *Guide to Local Production of WHO-Recommended Handrub Formulations* [28]. Furthermore, the choice of using alternative rheology modifiers in the gel formulations had the aim to investigate thickening agents naturally derived and poorly processed and to formulate sanitizers that would meet the common interest for eco-friendly and sustainable products.

## 2. Materials and Methods

### 2.1. Study Design

This trial was conducted from February to July 2020 and the experimental design sought to vary the WHO Formulation 1 [28] with two different gel formulations, trying to maintain the antimicrobial activity and improving the formulation in order to obtain efficient hand sanitizers, suitable for long-term use. In particular, Formulation 1 was used as control and as the starting-point formulation for the production of the two experimental gels containing natural extracts and viscosity enhancer excipients.

The products were arranged for the assessments of the stability tests (T_0_, T_7_, T_15_, one day of preparation, seven days of storage, and fifteen days of storage), in vitro and in vivo experiments.

### 2.2. Formulation

Three sanitizer products were prepared following the good manufacturing practices (GMPs) ISO 22716, by mixing different ingredients and in different percentages, as follows:

i. P1: this product corresponded to Formulation 1 [28] and its composition is of ethanol 96%, glycerol, hydrogen peroxide, and distilled water (80.0, 1.45, 0.125, 18.425% *v*/*v*);
ii. P2: this product was formulated with the following percentages (m/m) of ethanol 96% (56%), distilled water (41.10%), glycerol (0.7%), Aloe vera gel (1%), calendula glycolic extract (0.3%), xanthan gum (xg) (0.5%), benzyl alcohol (0.1%), lemon essential oil (0.15%), lavender essential oil (0.15%);
iii. P3: this product formulation was a modified version of the P2 formulation, in which the viscosity enhancer was changed from xanthan gum to high-viscosity hydroxyethyl cellulose, and certain ingredients in the concentration were modified as the following composition (m/m%) of ethanol 96% (60%), distilled water (38.4%), glycerol (0.5%), high-viscosity hydroxyethyl cellulose (1%), Aloe vera gel (0.15%), calendula glycolic extract (0.15%), benzyl alcohol (0.1%), lemon essential oil (0.12%), lavender essential oil (0.15%).

The products were collected in sterilized containers for subsequent analysis.

### 2.3. Evaluation of Hand Sanitizer Gels Stability

After the production, three independent replicates for each product, 27 samples in total, of 125 g were prepared in order to evaluate organoleptic properties, pH, alcohol strength by volume, and viscosity including the rheological behavior [29] before (chemical and physical analysis at T_0_) and after stability (accelerated storage tests) at temperatures of 20 ± 2 °C, 0 ± 2 °C, and 54 ± 2 °C until 15 days. At every endpoint, the organoleptic properties, pH, alcohol strength by volume, and viscosity were measured.

For the evaluation of the organoleptic properties, a sensorial analysis was conducted; more specifically, a visual analysis of the “appearance”, “color”, and an olfactory investigation of “odor.”

For the pH analyses, a benchtop pH meter (XS pH 8 + DHS Basic—Giorgio Bormac srl) was used, properly calibrated with three buffer solutions (pH 4, 7, and 10), and the temperature was set at 20 ± 2 °C during the test.

The values of the alcoholic strength by volume were measured through a precise density method (ISO 1183-1:2004, ASTM D854), using a pycnometer (0 dba—Gay Lussac) that enables the density of a fluid to be measured accurately by reference to an appropriate working fluid such as water or mercury, using an analytical balance.

In this study, the pycnometer was first filled with distilled water and weighted at the analytical balance at a constant temperature. According to the equation of density, the volume of water that filled the pycnometer and the stopper was as follows:

V = m_H2O_/ρ_H2O_ (1)

where m_H2O_ was the experimentally determined weight of water (empty pycnometer weight subtracted). After this, the procedure was repeated for our products (X) that had an unknown density (ρ_X_), and its weight m_L_ was determined (measured weight minus weight of empty pycnometer). Volume V obtained in this measurement was measured with the same equation used for water as

V = m_X_/ρ_X_ (2)

Combining Equations (1) and (2) m_H2O_/ρ_H2O_ = m_X_/ρ_X_ a relation that provides the density of measured products ρ_L_ was used ρ_X_ = (m_X_/m_H2O_)ρ_H2O_.

The values obtained for the products’ relative densities were checked in the tables approved by the World Organization of Legal Metrology, from which the value of ethylic alcohol in volume percentage was obtained.

For the evaluation of viscosity and rheological behavior of P2 and P3, a rotational viscometer (MyrVR 3000—Viscotech Hispania, SL, EI Vendrell, Spain) was used, and the result was a direct reading of the viscosity value in mPas (in this study, for simplification in the analysis of the results, viscosity was always converted in Pas). First, the viscosity was evaluated at 10 rpm (rotation per minute), which corresponded to a shear rate of 12.24 s^−1^ (User Manual Myr Viscometer Version 7.09). Taking into consideration the hydrocolloids examined, the R4 spindle was chosen. After this initial comparison, the rheological behavior of the two formulations was evaluated. To this aim, products were tested by measuring the viscosity on increasing shear rates (viscometry test) to study the effect of shear rate on viscosity. Specifically, five shear rates were applied: 5 rpm (6.20 s^−1^) 10 rpm (12.24 s^−1^), 30 rpm (36.72 s^−1^), 60 rpm (73.44 s^−1^), and 100 rpm (122.40 s^−1^). Every measurement was taken three times, and a mean value was calculated.

#### Accelerated Storage Tests

To assess the stability of a formulated product, it is usually exposed to “high stress,” i.e., the conditions of temperature, humidity, and light intensity, which cause break down. High-stress conditions enhance the deterioration of the product and hence reduce the time required for testing.

The three hand sanitizer products were subjected to high and low temperatures; in particular, two accelerated storage tests were conducted, according to European Chemicals Agency (ECHA) “Guidance on the Biocidal Products Regulation”, 2016.

- Cold Stability Testing: The test was conducted by storing the samples at 0 ± 2 °C, and the samples were checked before the test (T_0_) and at the endpoint T_7_;
- Hot Stability Testing: The test was conducted by storing the samples at 54 ± 2 °C, and the samples were checked before the test (T_0_) and at the endpoint T_15_.

In addition to the analysis listed before, whether syneresis (that is, the extraction or expulsion of a liquid from a gel) had occurred or not was checked and whether there was a presence of crystalline residues after the cold stability testing.

### 2.4. In Vitro Biocide Efficiency

Biocide efficacy was evaluated in vitro following the bactericidal activity phase 2/step 1 Quantitative Sensory Testing (QST) test, according to EN1276:1997, and the fungicidal activity phase 2/step 1 QST test, according to EN1650:1997, (ECHA Guidance on the Biocidal Products Regulation, 2016).

Regarding EN 1276, four different bacterial strains were exposed to the test substance—*Staphylococcus aureus* (*S. aureus*) ATCC 6538, *Enterococcus hirae* (*E. hirae*) ATCC 10541, *Escherichia coli* (*E. coli*) ATCC 10536, and *Pseudomonas aeruginosa* (*P. aeruginosa*) ATCC 15442; regarding the EN 1650, two different fungi strains—*Candida albicans* (*C. albicans*) ATCC 10231 (yeast) and *Aspergillus niger* (*A. niger*) ATCC 16404 (mold)—were exposed to the test substance.

The test substance was mixed with 1 mL of the test suspensions containing the different bacterial and fungi suspensions of known viability and the contact times were 1 and 5 min for bacteria strains and 1, 5, and 15 min for fungi strains; the temperature was maintained at 20 ± 2 °C for all the time of testing. After this contact time, 1 mL of the test mixture was immediately neutralized using 9 mL of LT 100 as the neutralizing agent in order to inactivate the killing activity and three different dilutions (from 10^−1^ to 10^−3^) of the test substance, made using tryptone water as the solvent, were plated in double in order to evaluate the viability reduction of the bacteria after exposure to the test substance.

Sabouraud dextrose agar (SDA) and tryptic soy agar (TSA) were used as nonspecific culture media, respectively for fungi and bacteria; plates were incubated at 37 ± 2 °C for 24 h for bacteria strains and for 48–72 h for fungi strains.

After the incubation time, viability reduction was calculated through the following formula:

R = (N × 10^−1^)/Na

where R = reduction of viability; N = bacterial counting for the initial test suspension (cfu/mL); N_a_ = bacterial counting for the test mixture at the end of contact time (cfu/mL).

The test substance was considered biocide when it caused a reduction of the vitality of at least 10^5^ at 20 ± 2 °C after 5 min contact for bacteria strains and after 15 min for fungi strains.

### 2.5. In Vivo Study

For this part of the study, eight volunteers were recruited to evaluate the acceptability, and tolerability of the three products and the impact that they may have on stratum corneum hydration. The study was a single-blind crossover trial based on a modified version of the WHO published protocol “Method for evaluation and comparison of tolerability and acceptability of different alcohol-based hand rubs: Method 2” [30]. In particular, the studies were conducted to investigate the impact on stratum corneum hydration and the hedonic evaluation.

The volunteers were recruited after the filling of a health questionnaire. After they were fully informed on the nature of the study and on the procedures involved, they gave their written consent. The participants did not suffer from any ailment and were not on any medication at the time of the study.

At the beginning of the trial, the control measurements (Control) were taken, both on the palm and on the back of the hands, before the distribution of the first product for volunteers to use. They used each of the three products exclusively for hand hygiene over a three-day period; a two-day washout period was imposed before switching from one product to the other. They did not use hand lotion or cream during the test periods.

The test periods and the data were considered usable by the observers when the participant used at least 30 g above 100 g of the product received, which corresponds to an application of 10 g per day. To this aim, each bottle distributed was weighed before and after the test period. Participants and observers’ meetings were planned one by one and always held in the same room, with the temperature always set at 25 ± 2 °C.

Epidermal hydration was assessed for the eight volunteers by measuring electrical capacitance with a chronometer (MoistureMeterSC—Delfin Technologies, Kuopio, Finland), an instrument that uses a measurement method based on the difference between the dielectric constant of water and other substances through measurement of the capacitance of a dielectric medium. Any change in the dielectric constant subsequent to the variation in skin surface hydration leads to an impaired calculated capacitance of a capacitor. This instrument contains two electrodes with different electrical charges that form an electromagnetic field that determines the dielectricity of the stratum corneum.

The probe is applied to the skin for 1 s at a pressure of 0.16 N. The measurement depth of the instrument varies and is determined by the thickness of the stratum corneum’s dry layer, thus acting as an individual parameter. The range of variation of the values of skin hydration degree is between 0 and 130 arbitrary units (AU). Values <35 are classified as very dry, values 35–50, as dry, and values >50 as normal. This technique makes skin hydration measurements extremely sensitive and reproducible.

In this case study, hydration was measured at a set temperature of 25 ± 2 °C, and every measurement was replicated three times.

Hydration values were measured both on the palm and on the back of the hand; left and right hands were investigated separately. The probe was decontaminated using an alcohol wipe before proceeding to the next participant.

The questionnaire concerning the hedonic evaluation was a modified version of the Questionnaire Part 2 of the WHO protocol [30]. In this questionnaire, volunteers were asked to evaluate different characteristics (color, smell, texture, irritation, drying effect, ease of use, speed of drying, application) of the three products on a scale from 1 to 7.

### 2.6. Data Analysis

Data were analyzed using factorial analysis of variance (ANOVA), using CoStat software (CoHort software, Monterey, CA, USA). The means were statistically separated on the basis of the Student–Newmann–Kewls test when the F test of ANOVA for treatment was significant at least at 0.05 probability level. Significance was accepted at *p* ≤ 0.05 level [31]. Regarding data on skin moisture content, it was interpreted following manufacturer’s instructions, right and left hand and palm and back of hands were all studied separately, and values were calculated and recorded as the mean of three measurements, and then they were statistically analyzed, as previously explained. Evaluation of tolerability and acceptability of the three ABHRs was performed according to the WHO protocol [9]. In Questionnaire Part 2, “Evaluation of the Test Product,” the items’ color and smell (fragrance) must score >50% above four in the seven-point scale, while all other items (texture, irritation, drying effect, etc.) must score >75% above four.

## 3. Results

### 3.1. Chemical and Physical Evaluation at T_0_

As shown in Table 1, the hand sanitizers had different organoleptic characteristics starting from appearance, smell, and color. These properties influenced the pleasantness of the product as perceived by the volunteers, application of the product, its persistence over time, and stability analyses.

The results showed that P1 had a value of ethylic alcohol in volume percentage higher than P2 and P3 but, despite the fact that it was prepared following Formulation 1 [28], actually showed a final value in alcohol strength of 79.22%; this could be related to alcohol evaporation during the procedure. At T_0_, the resulting pH values of the three hand sanitizers were statistically different from each other, with the lowest pH value of P1 due to the fact that it was the only formulation containing hydrogen peroxide. This value was even too acidic for the skin and, according to Ningsih et al. [32], could cause skin irritation. Instead, the values of P2 and P3 were understandable considering both the results of alcohol strength by volume and the ingredients’ percentage. However, it seems that, when considering pH, formulations 2 and 3 are more suitable as products to be applied to the skin; although the pH values should be in the skin’s pH range of 4.5–6.5 [33], Hasym and Baharudin (2011) reported that hand sanitizers with a pH > 6.5 could also be tolerated by the skin.

**Figure 1:**
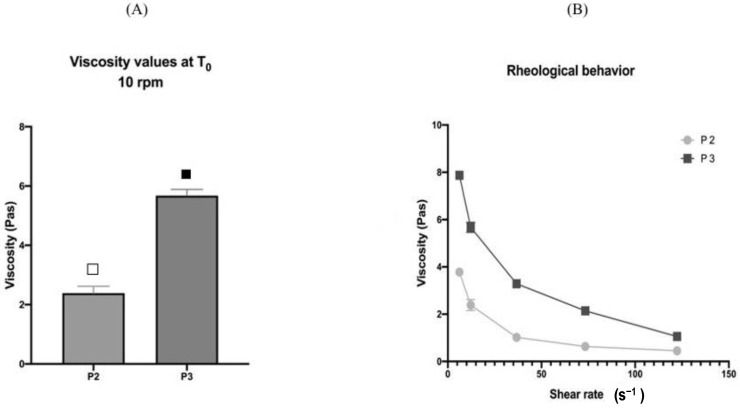
(**A**) Viscosity values at T_0_ with 10 rpm applied. (**B**) Rheological behavior at different shear rates applied. Values are expressed as the mean (±SD) of sample replicates (*n* = 3). Different symbols indicate the differences among the viscosity values on the two products (*p* ≤ 0.05).

As far as the viscosity values are concerned, viscosity was first measured at 10 rpm, which corresponded to a shear rate of 12.24 s^−1^; the results were calculated as the mean of three sample measurements and are represented in Figure 1A. P3 showed a higher viscosity, which was statistically different from P2 (respectively, 5.67 Pas and 2.38 Pas), and also resulted in a different appearance. Indeed, P2 appeared more liquid than P3, which, instead, had a typical gel appearance. This was related to the different thickeners used (XG for P2 and HEC for P3).

Hydroxyethyl cellulose and xanthan gum have been both poorly investigated regarding their behavior in hydroalcoholic gels. It is known that xanthan gum tends to be soluble in alcohol until 50% concentration and starts to be insoluble at higher concentrations of alcohol [34]. However, in this case study, xanthan gum showed an acceptable behavior as a thickening agent: in fact, using a concentration of just 0.5% m/m, a stable gel formulation was obtained. Nevertheless, as previously stated, in terms of appearance, P2 was more liquid than P3, which influenced also the hedonistic evaluation of volunteers.

Regarding P3, hydroxyethyl cellulose seemed to be a good thickening agent for the hydroalcoholic solution, with good results in viscosity and appearance. Indeed, the values of viscosity met the standard for a good hand sanitizer gel of 4.7–15.0 Pas [35], and also the appearance met the standard for a good gel, being no cloudy at all. Conversely, according to the study of Berardi and colleagues [4], HEC gel formulations containing >60% alcohol are not recommended due to the poor solubility of the polymer and the resulting cloudy appearance.

The different results presented in this study can be related to the type of HEC used for the preparation of the gel. In fact, there are 10 types of HEC available commercially, and the one used for the preparation of P3 is the high-viscosity HEC whose viscosity ranges between 0.8 and 5 Pas.

Rheological behavior was also investigated, and the results are shown in Figure 1B. P2 and P3 were tested by measuring the viscosity values on increasing shear rate (viscometry test) to study the effect of shear rate on viscosity. As expected, both the products behaved as “non-Newtonian fluids,” exhibiting a shear-thinning (or pseudo-plastic) behavior, as typical of polymeric dispersions. In fact, the viscosity decreased as the shear rate increased.

### 3.2. Accelerated Storage Tests

The results showed that at every endpoint, all the products were stable for odor and color, and just the appearance of the two gel formulations resulted differently. In fact, after hot storage, both appeared more liquid, especially P2.

The presence of crystalline residues after cold storage was not noted in P2 or P3. No *syneresis* occurred in P2 or in P3 after both the stability tests. Consequently, the three formulations were evaluated as stable for their organoleptic properties.

**Figure 2:**
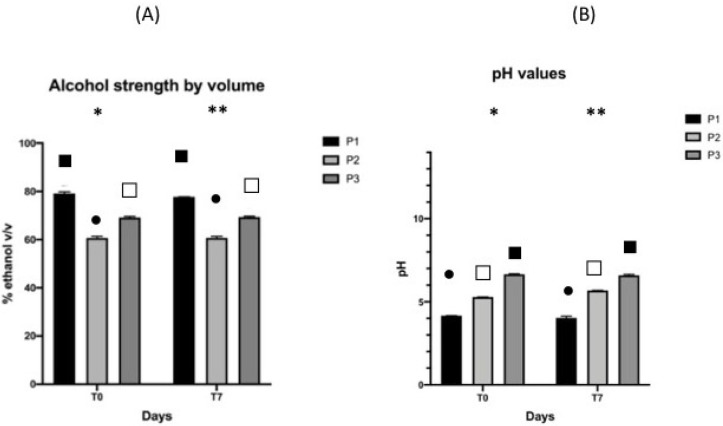
Alcohol strength by volume (**A**) and pH (**B**) before and after 7 days of cold storage at 0 ± 2 °C. Values are expressed as the mean (±SD) of sample replicates (*n* = 3). (∗) and (∗∗) indicate the difference among the products at the two endpoints, T0 and T7 respectively. Different symbols indicate the differences among the values in the three products (*p* ≤ 0.05).

**Figure 3:**
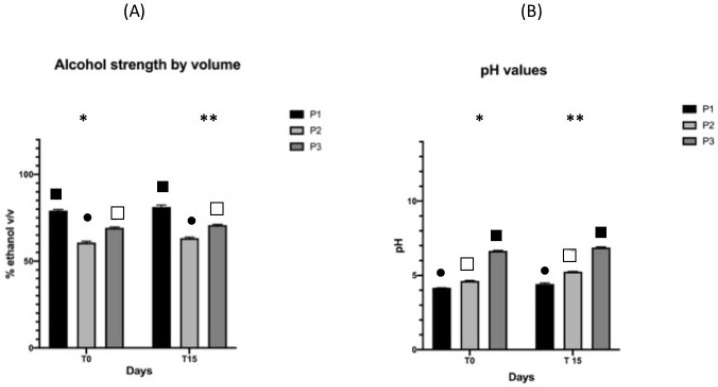
Alcohol strength by volume (**A**) and pH (**B**) before and after 15 days of hot storage at 52 ± 2 °C. Values are expressed as the mean (±SD) of sample replicates (*n* = 3). (∗) and (∗∗) indicate the difference among the products at the two endpoints, T0 and T7 respectively. Different symbols indicate the differences among the values in the three products (*p* ≤ 0.05).

The results of the alcohol strength by volume and pH after Cold Stability Testing at 0 ± 2°C and after Hot Stability Testing at 52 ± 2 °C are shown in Figure 2 and Figure 3, respectively.

Statistical studies showed that after cold and hot storage, there was an overall significant decrease in the alcohol strength values and an increase in the pH values after both storages. The connection between the two changes is understandable considering that a lower concentration of ethanol makes the pH more alkaline. Nevertheless, we were not able to provide an explanation of the overall behavior, and more investigation should be carried out. Despite these differences, it is reasonable to claim that overall, for pH and alcohol strength, the products can be considered stable; indeed, the values were kept in an acceptable range after both storages.

**Figure 4:**
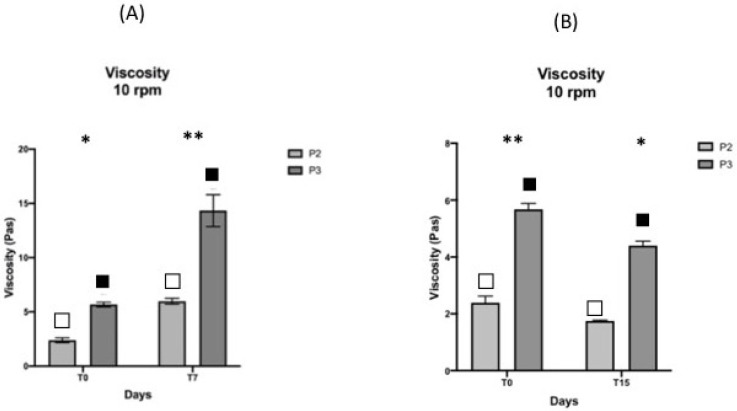
Viscosity values before and after storage for 7 days at 0 ± 2 °C (**A**) and before and after hot storage for 15 days at 52 ± 2 °C (**B**). Values are expressed as the mean (±SD) of sample replicates (*n* = 3). (∗) and (∗∗) indicate the difference among the products at the two endpoints, T0 and T7 respectively. Different symbols indicate the differences among the values in the two products (*p* ≤ 0.05).

With regard to viscosity, it is known that it strongly depends on the temperature; in fact, liquid and hydrocolloid viscosity usually decreases with increasing temperature and the opposite for decreasing temperature. This was confirmed by the two products investigated. As the graphs in Figure 4 show, statistically, there was a significant difference between the values at T_0_ and those after both cold and hot storages.

### 3.3. In Vitro Biocide Efficacy

The results in terms of reduction of viability after the two tests bactericidal activity phase 2/step 1 QST test, according to EN1276:1997, and fungicidal activity phase2/step 1 QST test, according to EN1650:1997, of P1, P2 and P3 are shown in Table 2.

According to EN 1276 and EN 1650 tests, a substance is considered biocide when it causes a reduction of the vitality of at least 10^5^ at 20 ± 2 °C after a maximum of 5 min contact for each bacteria strain and after a maximum of 15 min contact for each fungi strain. From the analysis, P1 resulted as the most efficient biocide, and this formulation could be considered 100% bactericidal and fungicidal after a contact time of 1 min.

Nevertheless, P2 proved to be also very efficient after a time of contact of 1 min in both tests; indeed, it failed the test at 1 min only for *P. aeruginosa* and *A. niger*, although these two pathogens were completely killed after 5 min. This efficiency could be attributable to the presence in the formulation of lemon and lavender essential oils, which have an antimicrobial efficacy [36], and it may also be related to the addition of Aloe vera; indeed, this one also seems to be efficient against some pathogens [37]. The fact that P2, even with a lower concentration of ethanol, proved efficient may also be a demonstration that sanitizers containing 60% to 95% alcohol are almost equally acceptable [7].

Despite our predictions, considering the different percentages of the ingredients, the results of tests on P3 were unexpected; indeed, P3 showed lower activity than P2 against *E. coli*, *E. hirae*, *P. aeruginosa*, *C. albicans*, and *A. niger* after a time of contact of 1 min. In addition, if P2 gave a 100% vitality reduction for all the strains after a time of contact of 5 min, with P3 after 5 min there was still growth (even if minimal and acceptable) of *E. coli*, *E. hirae*, and *A. niger*.

These differences can be explicated by shifting the attention from the concentration of ethanol and considering all the formulations. As shown in the formulation paragraph, P3 contains a lower concentration of water, essential oils, and Aloe vera than P2; as stated previously, essential oils and Aloe vera are effective in performing an antimicrobial activity, and thus, their reduction in concentration can be a reason for the lower activity of P3. Another reason may be found in the different concentrations of water; indeed, according to Block [8], pathogens proteins are not denatured easily by ethanol with a lower presence of water.

### 3.4. In Vivo Study

One main issue concerning the frequent use of ABHRs is irritant contact dermatitis and skin deterioration, which lead to skin irritation, skin dryness, enlargement of the pores, thus making sensitive skin more susceptible to infection [16].

According to Ahmed-Lecheheb et al. [38], HCWs sometimes have low compliance with hand hygiene because of the assumption that it will lead to skin irritation, dryness, and hand eczema. In a prospective intervention trial designed and conducted by Girard et al. [39] to study the impact of ABHRs use on hand hygiene compliance among HCWs, dermatologist-assessed skin dryness and irritation showed that the ABHRs was better tolerated than the traditional antiseptic handwashing preparation. Nevertheless, repeated exposure to alcohol can cause or maintain skin dryness and irritation [38].

Thus, in an overall study about different hand sanitizer formulations, it is important to consider the impact of the product on the stratum corneum and the compliance of individuals [14]. In this case study, the moisture content of the stratum corneum before and after the treatment with each product was investigated.

There are large variations in skin hydration across different anatomical areas, even when studying the hand; it is important to consider separately the palm and the back of the right and left hand [40].

**Figure 5:**
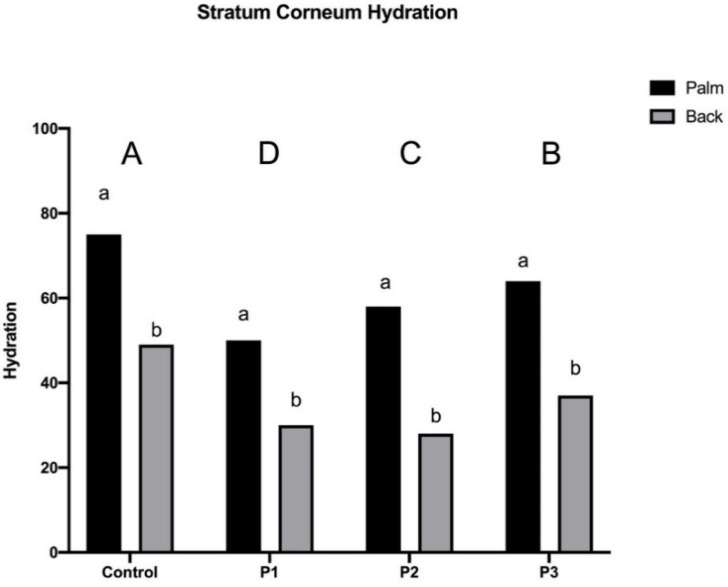
Mean (*n* = 8) of front and back hydration of different volunteers in response to three products at 3 days after treatment. Values are expressed as mean of measurement replicates (*n* = 3). Different uppercase letters indicate statistically significant differences among the hydration in all different products’ treatments. Different lowercase letters indicate statistically significant differences among the hydration in the single product (*p* ≤ 0.05).

As previously stated, right and left hand and palm and back of hand were all studied separately, and the results showed that all the products were statistically different from each other and to the Control. However, it was also discovered that there was no significant difference between right- and left-hand measurements for each product. The results were, in fact, statistically significant only in a comparison between palm and back (both seen as a mean value of the right and left back of the eight volunteers), and only these measurements were elaborated. The results are shown in Figure 5.

It was reported by Harbarth et al. [41] that hand gel containing an increased glycerin concentration and 70% ethanol can increase skin hydration. Nevertheless, in this case study, P1 caused a decrease in hydration after three days of use. In particular, there was a decrease of 25.63 ± 6.49 on the palm and 20.11 ± 18.52 on the back. Indeed, even if glycerol was added as a humectant in the formulation, no more emollients were included, and 80% *v/v* of ethanol and 0.125% of hydrogen peroxide were poorly tolerated by the skin.

Conversely, after the use of P2 and P3, the hydration increased, which may be related to the lower concentration of ethanol, the absence of hydrogen peroxide, and the addition of glycerin and other emollients such as Aloe vera and *Calendula officinalis* to the formulation. Comparing P2 and P3, the only difference related to the back of the hand. After three days of application, P2 increased the mean hydration of the palm to 8.25 ± 4.51, but there was a decrease, even if really low, of 2.05 ± 5.17 on the back. Regarding P3, hydration of the palm and the back, respectively, amounted to 6.35 ± 3.57 and 8.22 ± 12.84. Nevertheless, in view of our results, the data on the palms are more valuable than that regarding the back of the hand for a hand product. Moreover, in many studies, the palm is the only area considered when evaluating skin hydration [40]. Furthermore, in P2 formulation, the concentrations of glycerin, Aloe vera, and *Calendula officinalis* were slightly higher than the ones in P3, and the percentage of ethanol was lower in P2 than in P3.

Consequently, when taking all this into account, it is reasonable to say that P2 and P3 are almost equal considering the impact on the stratum corneum hydration, and for a better comparison, they should be further investigated with the evaluation of other parameters, for instance, the Trans Epidermal Water Loss (TEWL), sebum content, and the pH of the skin.

**Figure 6:**
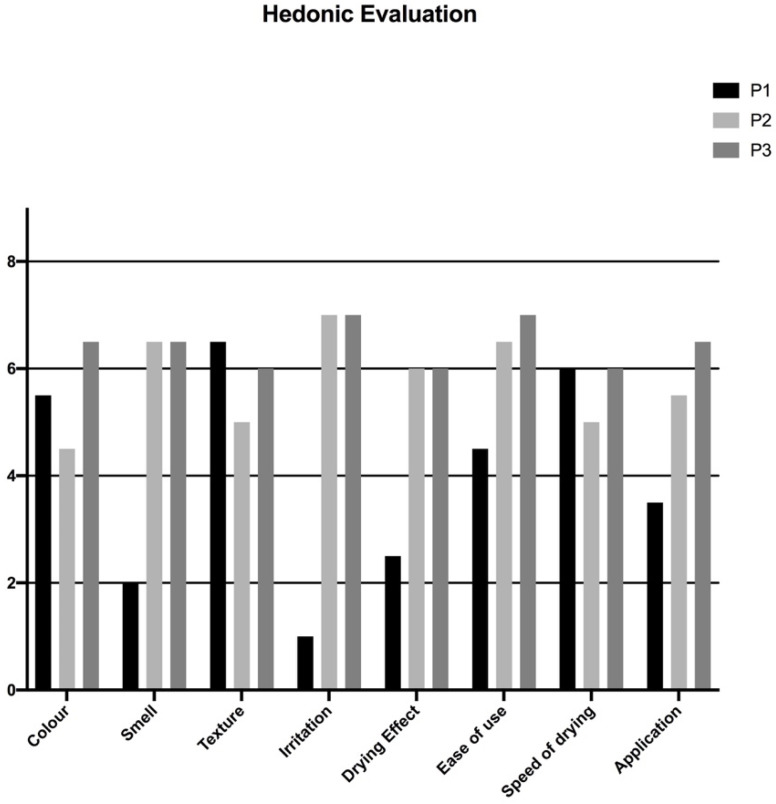
User acceptability for each of the alcohol-based hand rub (ABHR) products tested.

For what concerns the hedonic evaluation of the three products, the questionnaires completed by the volunteers for each product were collected, and the elaborated results are shown in Figure 6.

Taking into consideration the parameters of the WHO Protocol [30], P1 resulted in “acceptable” just for the color, with a score higher than 4 on 100% of volunteers, and “not acceptable” for all the other items. Thus, overall, the product is considered “not acceptable”.

P2 resulted in “acceptable” for smell, irritation, drying effect, ease of use, and application with a score higher than 4, respectively, on 87.5%, 100%, 100% of volunteers. Additionally, it resulted in “not acceptable” for color, texture, and speed of drying due to the results of the dull yellow color and the fact that it was more liquid than typical gel formulations. Thus, we may consider the product “acceptable”, but some modifications can be proposed to improve its acceptability.

P3 resulted in “acceptable” for all the characteristics evaluated with a score higher than 4, respectively, on 87.5% and 100% of volunteers, and thus, it can be considered the best product of the three discussed.

This study had some limitations. The WHO protocol [30] was designed for healthcare workers, but it was not possible to use randomization and double blinding in our study due to the limitations of the setting. Moreover, the WHO protocol requests a group of 40 volunteers, and in this study, only eight volunteers were recruited. Nevertheless, our first aim was not to find a product to be used as the first choice in hospitals or other health fields (that is usually the aim of WHO Protocol) but only to formulate and evaluate two sanitizer products that can be simultaneously efficient, tolerable, and acceptable and to compare them with the WHO Formulation 1.

## 4. Conclusions

In this study, the importance of an extensive and thorough evaluation of hand sanitizer products is highlighted. P1 was obviously found to be efficient as a biocide, but it was also found to be poorly tolerated by the skin through hydration measurements. In addition, it was not considered acceptable or tolerable in volunteer hedonic values, especially in terms of irritation and drying effect, with an expectable reduction in compliance. Furthermore, its liquid formulation limits its use due to the spillage and its difficulty to use in portable dosage forms and may increase the alcohol evaporation rate.

From the chemical and physical analysis point of view, P2 and P3 proved to be both good products regarding pH and alcohol strength values. However, in terms of viscosity, P3 seemed to be a better gel product than P2, and this was also confirmed by the hedonic evaluations in terms of texture, ease of use, and application. Moreover, they were well tolerated by the skin, increasing the hydration of the stratum corneum, due to the addition in the formula of natural emollients as *Calendula officinalis* and Aloe vera. Despite a lower ethanol concentration than P1, P2 and P3 also showed an acceptable biocide efficiency, with better results in P2.

In conclusion, it is reasonable to state that gel formulations are easier to use than liquid ones and can increase compliance. Moreover, in a period when the use of ABHRs is more frequent, for instance, among healthcare workers or during a pandemic, it is important to find and select products that have a good balance between effectiveness, safety, and compatibility with the skin, and that are acceptable to the users. Thus, in our opinion, it would seem that both P2 and P3 can respond to this need; moreover, it would be useful to continue the trials with the aim to test the optimum ratio of alcohol to emollients (with the minimum possible amount of alcohol) that provides the best antimicrobial action.

## Figures and Tables

**Table 1:** The organoleptic properties and pH and alcohol strength by volume values of the three sanitizer products at T_0_. Every value was calculated as the mean (±SD) of three sample measurements (*n* = 3). The different letters (^a^, ^b^, and ^c^) represent significant differences between the products (*p* ≤ 0.05).

Products	Colour	Odor	Appearance	pH	% Ethanol *v*/*v*
P1	Transparent	Characteristic of ethanol	Liquid	4.16 ± 0.0057 ^c^	79.22 ± 0.6080 ^a^
P2	Dull yellow	Characteristic of lemon essential oil	Gel	5.29 ± 0.0057 ^b^	60.69 ± 0.6870 ^c^
P3	Opaque transparent	Characteristic of lemon essential oil	Gel	6.65 ± 0.0321 ^a^	69.17 ± 0.470 ^b^

**Table 2:** Biocide efficacy for P1, P2, P3 after 1, 5, and 15 min of contact with the pathogen. Every result is calculated as the mean of the counts of two plates.

Products	Contact Time	*S. aureus*		*E. coli*		*E. hirae*		*P. aeruginosa*		*C. albicans*		*A. niger*	
		Growth (cfu/mL)	Vitality Reduction	Growth (cfu/mL)	Vitality Reduction	Growth (cfu/mL)	Vitality Reduction	Growth (cfu/mL)	Vitality Reduction	Growth (cfu/mL)	Vitality Reduction	Growth (cfu/mL)	Vitality Reduction
**P1**	***Inoculum***	1.60 × 10^7^		1.54 × 10^9^		3.65 × 10^8^		3.30 × 10^9^		1.14 × 10^7^		2.85 × 10^7^	
	1 min.	0	1.60 × 10^7^ (100%)	0	1.54 × 10^9^ (100%)	0	3.65 × 10^8^ (100%)	0	3.30 × 10^9^ (100%)	0	1.14 × 10^7^ (100%)	0	2.85 × 10^7^ (100%)
	5 min.	0	1.60 × 10^7^ (100%)	0	1.54 × 10^9^ (100%)	0	3.65 × 10^8^ (100%)	0	3.30 × 10^9^ (100%)	0	1.14 × 10^7^ (100%)	0	2.85 × 10^7^ (100%)
	15 min.	N/A	N/A	N/A	N/A	N/A	N/A	N/A	N/A	0	1.14 × 10^7^ (100%)	0	2.85 × 10^7^ (100%)
**P2**	***Inoculum***	1.60 × 10^7^		1.54 × 10^9^		3.65 × 10^8^		3.30 × 10^9^		1.14 × 10^7^		2.85 × 10^7^	
	1 min.	0	1.60 × 10^7^ (100%)	0	1.54 × 10^9^ (100%)	0	3.65 × 10^8^ (100%)	1.00 × 10^5^	3.30 × 10^3^ (99.9%)	0	1.14 × 10^7^ (100%)	5.00 × 10^3^	5.70 × 10^2^ (99%)
	5 min.	0	1.60 × 10^7^ (100%)	0	1.54 × 10^9^ (100%)	0	3.65 × 10^8^ (100%)	0	3.30 × 10^9^ (100%)	0	1.14 × 10^7^ (100%)	0	2.85 × 10^7^ (100%)
	15 min.	N/A	N/A	N/A	N/A	N/A	N/A	N/A	N/A	0	1.14 × 10^7^ (100%)	0	2.85 × 10^7^ (100%)
**P3**	***Inoculum***	1.60 × 10^7^		1.54 × 10^9^		3.65 × 10^8^		3.30 × 10^9^		1.14 × 10^7^		2.85 × 10^7^	
	1 min.	0	1.60 × 10^7^ (100%)	1.48 × 10^5^	1.04 × 10^3^ (99.9%)	1.36 × 10^7^	2.68 × 10^1^ (90%)	2.87 × 10^7^	1.14 × 10^1^ (90%)	1.00 × 10^4^	1.14 × 10^2^ (99%)	1.70 × 10^5^	1.65 × 10^1^ (90%)
	5 min.	0	1.60 × 10^7^ (100%)	5.00 × 10^2^	3.08 × 10^5^ (99.9%)	3.00 × 10^3^	1.21 × 10^4^ (99.9%)	0	3.30 × 10^9^ (100%)	0	1.14 × 10^7^ (100%)	2.50 × 10^3^	1.14 × 10^3^ (99.9%)
	15 min.	N/A	N/A	N/A	N/A	N/A	N/A	N/A	N/A	0	1.14 × 10^7^ (100%)	0	2.85 × 10^7^ (100%)

## Data Availability

The data used to support the findings of this study are available from the corresponding author upon request.
